# Cyclic AMP Pathway Activation and Extracellular Zinc Induce Rapid Intracellular Zinc Mobilization in *Candida albicans*

**DOI:** 10.3389/fmicb.2018.00502

**Published:** 2018-03-21

**Authors:** Lasse Kjellerup, Anne-Marie L. Winther, Duncan Wilson, Anja T. Fuglsang

**Affiliations:** ^1^Department of Plant and Environmental Sciences, University of Copenhagen, Frederiksberg, Denmark; ^2^Pcovery ApS, Copenhagen, Denmark; ^3^Medical Research Council Centre for Medical Mycology, University of Aberdeen, Aberdeen Fungal Group, Aberdeen, United Kingdom

**Keywords:** cAMP, zinc, *Candida albicans*, signaling, ER

## Abstract

Zinc is an essential micronutrient, required for a range of zinc-dependent enzymes and transcription factors. In mammalian cells, zinc serves as a second messenger molecule. However, a role for zinc in signaling has not yet been established in the fungal kingdom. Here, we used the intracellular zinc reporter, zinbo-5, which allowed visualization of zinc in the endoplasmic reticulum and other components of the internal membrane system in *Candida albicans*. We provide evidence for a link between cyclic AMP/PKA- and zinc-signaling in this major human fungal pathogen. Glucose stimulation, which triggers a cyclic AMP spike in this fungus resulted in rapid intracellular zinc mobilization and this “zinc flux” could be stimulated with phosphodiesterase inhibitors and blocked via inhibition of adenylate cyclase or PKA. A similar mobilization of intracellular zinc was generated by stimulation of cells with extracellular zinc and this effect could be reversed with the chelator EDTA. However, zinc-induced zinc flux was found to be cyclic AMP independent. In summary, we show that activation of the cyclic AMP/PKA pathway triggers intracellular zinc mobilization in a fungus. To our knowledge, this is the first described link between cyclic AMP signaling and zinc homeostasis in a human fungal pathogen.

## Introduction

Zinc is an essential micronutrient for all living organisms and maintaining precise zinc homeostasis is vital for cell survival. Indeed, in eukaryotes, 9% of the proteome requires zinc (Andreini et al., 2009). Zinc proteins can be categorized in four groups: enzymes (47%), transcription factors (44%), storage and transport (5%), and cell signaling (3%). Zinc is used as a co-factor or structural component in more than 300 enzymes within all 6 classes (Bozym et al., 2006).

*Candida albicans* is an opportunistic fungal pathogen and a major cause of invasive fungal infections, where mortality can reach 40% (Kullberg and Arendrup, 2015), and of vulvovaginal candidiasis, which affects 75% of women of childbearing age (Brown et al., 2012).

Metals, such as zinc, play important roles in the control of microbial infections. Depending on the host niche, the immune system can attempt to restrict microbial access to zinc, or to expose them to potentially toxic levels. Collectively these processes are known as “nutritional immunity” (Hood and Skaar, 2012). The mammalian protein calprotectin is used to decorate neutrophil extracellular traps where it elicits anti-*Candida* activity via zinc chelation (Urban et al., 2009). On the other hand, *C. albicans* can produce its own secreted zinc-binding protein Pra1 in order to scavenge this metal from host tissue (Citiulo et al., 2012).

In mammalian cells zinc is emerging as an important intracellular second messenger (Yamasaki et al., 2007). Zinc signals can be classified as either early, which occur within minutes, or late signals, which are transcription dependent (Fukada et al., 2011). The early signals are dependent on release of zinc from proteins or via transporter-mediated release from intracellular organelles. Eukaryotes, including both fungi and metazoans have two known families of zinc-transporters. The Zrt/Irt-like protein (ZIP) family imports zinc from outside of the cell or from within organelles into the cytoplasm. Although the first ZIP structure has recently been reported (Zhang et al., 2017), the precise transport mechanism remains unclear (Eide, 2006). In contrast, members of the cation diffusion facilitator (CDF) family can transport zinc from the cytosol, either sequestering it within organelles or exporting to the extracellular environment. These CDF or ZnT transporters functions as H^+^ or K^+^ antiporters (Eide, 2006). In mammalian cells, a phenomenon called the zinc wave has been described whereby activation of the FcεRI receptor leads to zinc release from the endoplasmic reticulum (ER) (Yamasaki et al., 2007). This ER export is mediated by the zinc transporter ZIP7, which is activated via phosphorylation by Protein Kinase 2 (CK2) (Taylor et al., 2012).

A role for zinc has been identified in several signaling cascades, such as in gastrula cells in zebra fish where STAT3 activation leads to activation of ZIP6, which increases the cytoplasmic zinc concentration. This leads to activation of the zinc finger transcription factor Snail, which regulates cell movement (Yamashita et al., 2004; Fukada et al., 2011). Additionally, in mast cells, Protein Kinase C (PKC) is regulated by zinc that is imported to the Golgi by the zinc transporter ZnT5 (Nishida et al., 2009; Fukada et al., 2011) and this PKC activation then leads to NF-κB mediated cytokine production.

Although zinc has not yet been described as an intracellular signaling molecule within the fungal kingdom, an ER zinc pool has been reported in several fungal species. In *Saccharomyces cerevisiae* two transporters belonging to the CDF family, Msc2 and Zrg17, have been shown to localize to the ER, where they function as heterodimers (Li and Kaplan, 2001; Ellis et al., 2005). Both Msc2 and zinc have been shown to be required for normal ER function, as low zinc or deletion of *MSC2* leads to the unfolded protein response (UPR), which is a hallmark of ER stress (Ellis et al., 2004). Deletion of the CDF family zinc transporter *ZHF* located to the ER in *Schizosaccharomyces pombe* leads to hypersensitivity to zinc and cobalt (Clemens et al., 2002).

Fluorescent probes have frequently been used to assess intracellular pools of zinc (Lim et al., 2004; Dean et al., 2012). Different probes can be used to study zinc in different intracellular compartments. The zinc probe zinquin has revealed the presence of so-called zincosomes in *S. cerevisiae* (Devirgiliis et al., 2004). These are vesicular-like bodies containing zinc, but their function remains unclear (Eide, 2006). Zinquin is able to interact with ligand bound zinc and is therefore most likely reporting on zinc bound to various zincosomal proteins (Wellenreuther et al., 2009). Vacuolar zinc in *S. cerevisiae* has been detected using the zinc probe FuraZin-1 (MacDiarmid et al., 2003). Zinbo-5, which is used in this study, has also previously been used to track changes in labile (free) zinc in yeast in response to compounds which alter zinc homeostasis (Simm et al., 2011). The localization of zinbo-5 in this previous study, however, was not examined.

Here we observed that zinbo-5 in *C. albicans* reports on non-vacuolar zinc, including the ER pool. Using this probe we have identified rapid and dynamic changes in intracellular zinc in response to stimulation with extracellular zinc as well as cyclic AMP/PKA pathway activation.

## Materials and Methods

### Strains and Growth Conditions

The laboratory *C. albicans* strain BWP17 + CIp30 (Mayer et al., 2012) was used for all experiments and maintained on YPD agar (1% yeast extract, 2% peptone, 2% glucose, 2% agar). For experiments, cells were grown overnight in liquid YPD or in SD medium [0.171% yeast nitrogen base (Sunrise Science Products), 2% glucose and 0.5% NH_4_SO_4_] as indicated.

### Microscopy

Cells from an overnight culture in YPD-media were washed in PBS buffer and diluted to an OD_600_ of 0.01. One hundred and fifty microliters cell plus 150 μL of 2× RPMI-1640 media (R6504, Sigma) was transferred to wells of an 8-well slide (80826, Ibidi). The cells were grown for 3.5–4.5 h at 37°C before washing three times in PBS buffer.

Cell staining was performed in 200 μL PBS within the 8-well slide. For zinbo-5 (sc-222425, Santa Cruz), 5 μM for 15 min; DiOC_6_(3) (D273, Thermo Fisher), 2 μM for 3 min; Syto13 (S7575, Thermo Fisher) 2 μM for 5 min; DAPI (D1306, Thermo Fisher) 5 μM for 10 min. For Syto13, cells were first incubated overnight in PBS at 37°C prior to staining. After staining, cells were washed in PBS. For pulse-chase staining of the fungal vacuole, cells were incubated in RPMI for 2 h, incubated with 5 μM FM4-64 in RPMI for 2 h (pulse), washed with PBS, incubated for a further 1 h in RPMI in the absence of dye (chase), washed and imaged. An inverted confocal laser microscope (Leica SP5, Leica Microsystems) equipped with a 63× water-immersion objective was used for obtaining fluorescent images of the stained cells. The following laser settings was used: zinbo-5 and DAPI, 405 nm excitation, 415–470 nm detection; DiOC_6_(3), 488 nm excitation, 495–620 nm detection; FM4-64, 513 nm excitation, 650–750 nm detection; Syto13, 488 nm excitation, 495–510 nm detection. The pictures were analyzed in the open source program ImageJ.

### Fluorescence Kinetics

Cells were grown overnight in SD media containing 10 μM ZnSO_4_, unless stated otherwise. The cells were washed in PBS buffer (D8537, Sigma) three times by centrifugation at 2000 *g* for 2 min and re-suspended to an OD_600_ of 1.8. One hundred microliters cells plus 50 μl of 20 μM zinbo-5 was mixed in a 96 well black plate and fluorescence measured on a plate reader (SpectraMax M2, Molecular Devices). Measurements was performed every 1 min with excitation at 405 nm, emission at 443 nm and emission filter at 420 nm. Twenty-five microliter zinc and EDTA were added to the final concentrations indicated. A four-parameter variable slope equation was performed in GraphPad Prism 6 to calculate the EC_50_ in the zinc titration experiment. Experiments with chemical inhibitors/activators of zinc fluxes were performed in 0.5 M MOPS-tris buffer pH 7.0 containing 5 μM EDTA to prevent changes in pH. The following compounds were used: 25 μl of dithiothreitol (DTT, D0632, Sigma), theophylline (T1633, Sigma), caffeine (C8960, Sigma), dibutyryl cAMP (D0260, Sigma), MDL12330 (sc-3537, Santa Cruz,) or myristoylated PKI (14–22) (myr-PKI, sc-471154, Santa Cruz,). Fifty micromolar clioquinol (33931, Sigma) was used as control for minimum zinbo-5 fluorescence.

For determination of metal selectivity, the following metals were added to a final concentration of 23 μM: ZnSO_4_, CuSO_4_, NiCl_2_, CoCl_2_, FeSO_4_, CdCl_2_, MgSO_4_, CaCl_2_.

*P*-values were calculated in one- or two-way analysis of variance (ANOVA) followed by Tukey (one-way) or Dunnett (two-way) *post hoc* testing in GraphPad Prism 6.

### Zinc Uptake Assay

Zinc uptake was measured either following overnight incubation in SD + 10 μM Zn^2+^ media (∼18 h) or after addition of a zinc pulse to cells washed and re-suspended in PBS buffer to OD_600_ of 1.0. For the zinc pulse, 5 μM ZnSO_4_ was added for 20 min at room temperature. Cells were pelleted and 25 μl supernatant transferred to 75 μl of 0.5 M MOPS-tris buffer pH 7.0 containing 0.2 M KCl and 0.66 μM cell impermeant FluoZin-3 (F24194, Thermo Fisher). The fluorescence was measured on a plate reader (SpectraMax M2, Molecular Devices) with excitation at 485 nm and emission at 525 nm. A standard curve of ZnSO_4_ was prepared in PBS and included with every experiment. The amount of zinc in the supernatant was found to fit a second order polynomial equation in Excel (Microsoft) from the ZnSO_4_ standard curves of the FluoZin-3 signal.

## Results

### Zinbo-5 Localizes to the Internal Membrane System Including the ER in *C. albicans*

**FIGURE 1:**
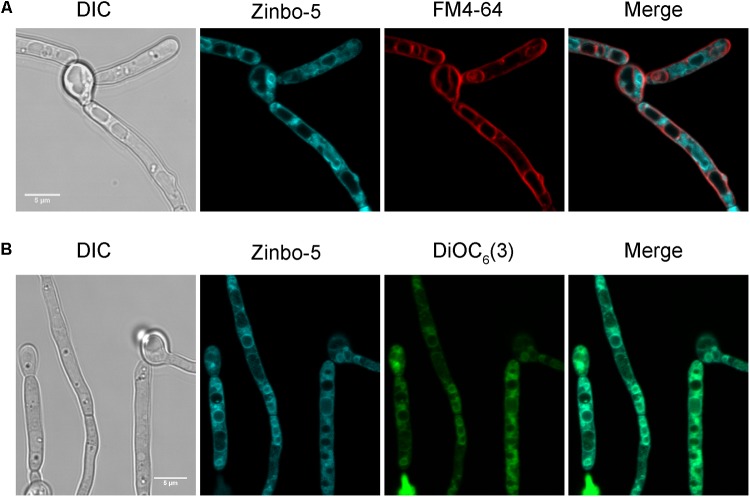
Zinbo-5 is excluded from the vacuole and localizes to the internal membrane system in *Candida albicans*. **(A)** Cells stained with zinbo-5 and FM4-64, which, under these conditions stains the plasma membrane and vacuolar membrane. **(B)** Co-localization of zinbo-5 and DiOC_6_(3) (internal membranes).

The cellular localization of the zinc probe zinbo-5 was first determined using fluorescence microscopy. Co-staining of *C. albicans* with the vacuolar membrane stain FM4-64 demonstrated that zinbo-5 is excluded from the vacuole (**Figure 1A**). The fungal vacuole can also be observed as an apparent indentation by differential interference contrast (DIC) (**Figure 1A**). Instead, zinbo-5 appeared to localize to non-vacuolar intracellular membrane structures. **Figure 1B** shows co-localization of zinbo-5 with DiOC_6_(3). At the concentrations used here, DiOC_6_(3) has been reported to specifically stain the nuclear envelope and ER in *S. cerevisiae* (Koning et al., 1993).

**FIGURE 2:**
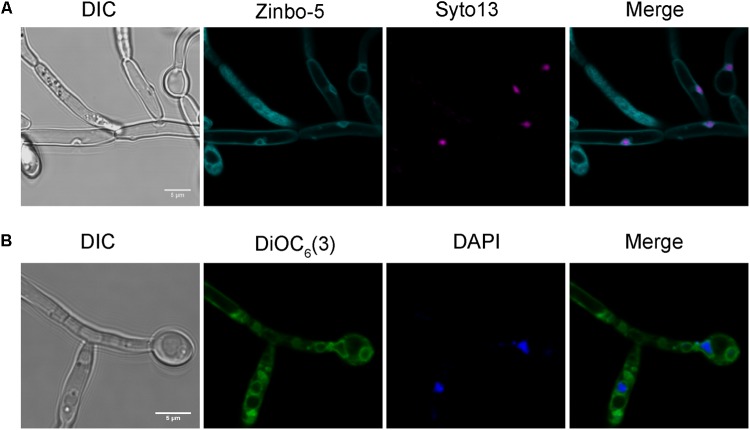
Zinbo-5 and DiOC_6_(3) localizes to the perinuclear endoplasmic reticulum. **(A)** Cells stained with zinbo-5 and the nuclear stain Syto13. Cells were first incubated in PBS buffer overnight at 37°C prior to staining with this dye. This incubation period also changed the zinbo-5 staining pattern to the plasma membrane and the nucleus. **(B)** Cells stained with DiOC_6_(3) and the nuclear stain DAPI.

We therefore investigated whether zinbo-5 associates with the nucleus in *C. albicans* using the nuclear dyes Syto13 and DAPI (**Figures 2A,B**). In order to stain the fungal nucleus with Syto13, cells were first incubated overnight in PBS. Interestingly, this incubation period altered zinbo-5 localization, possibly due to changes in the cells’ internal membrane system or redistribution of the intracellular zinc pool during this extensive period in buffer. Under these conditions, zinbo-5 fluorescence localized as a ring surrounding the nucleus, likely to be the perinuclear ER (**Figure 2A**), as did DiOC_6_(3) (**Figure 2B**). Therefore, in *C. albicans* zinbo-5 appears to stain the internal membrane system, including the perinuclear ER and most likely also other parts of the ER.

### Intracellular Zinc Is Highly Dynamic and Responds to Extracellular Zinc

**FIGURE 3:**
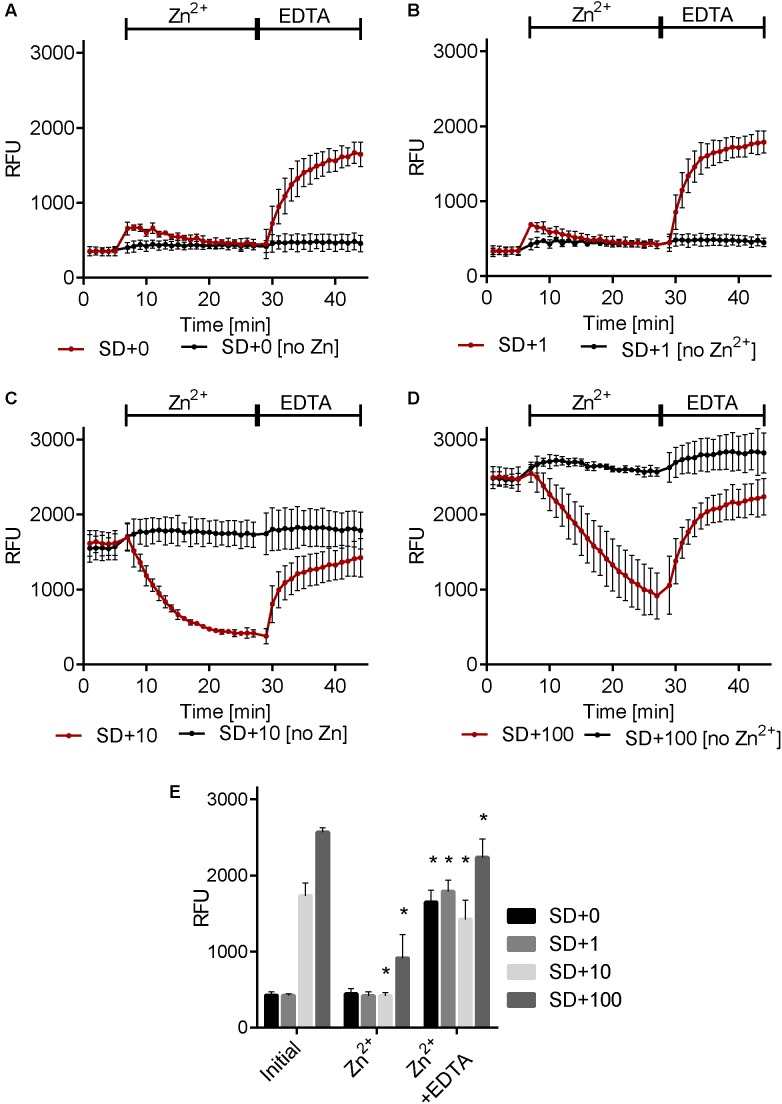
Environmental Zn^2+^ induces dynamic changes in the cellular zinc pool. Cells were grown in SD media containing 0 μM Zn^2+^ [SD+0 **(A)**], 1 μM Zn^2+^ [SD+1 **(B)**], 10 μM Zn^2+^ [SD+10 **(C)**] or 100 μM Zn^2+^ [SD+100 **(D)**]. Zinbo-5 was added and the zinbo-5 fluorescence determined following 10 min of incubation with probe (initial), followed by 20 min incubation with 5.7 μM zinc (Zn^2+^) and following a subsequent 15 min incubation with 50 μM EDTA (Zn^2+^ + EDTA). **(E)** Shows a composite of single measurements of the different conditions from **A–D** at the beginning of the experiment (initial), and following 20 min zinc treatment (Zn^2+^), followed by 15 EDTA treatment (Zn^2+^ + EDTA). Error bars indicate standard deviation for *n* = 3. “^∗^” indicate *p* < 0.01 in two-way ANOVA test for the treatment compared to before treatment (‘Zn^2+^’ compared to ‘initial’ and ‘Zn^2+^ + EDTA’ compared to ‘Zn^2+^’).

In mammalian cells, it has been demonstrated that stimulation with extracellular zinc results in the release of zinc from intracellular stores. Using zinbo-5 as a reporter, we tested whether a similar phenomenon occurs in fungi. *C. albicans* cells were pre-grown in minimal medium (SD) supplemented with 0, 1, 10, or 100 μM Zn^2+^. **Figure 3** shows that the initial zinbo-5 signal intensified with increased zinc in the culture medium. Cells were then stimulated with 5.7 μM exogenous Zn^2+^ for 20 min. Interestingly, cells pre-cultured in the presence of (≥10 μM) Zn^2+^ exhibited a rapid loss of zinbo-5 fluorescence (**Figure 3**). Extracellular Zn^2+^ was then sequestered via addition of the chelator EDTA for 15 min. This treatment resulted in an increase in zinbo-5 signal for all samples. Cells that were not stimulated with Zn^2+^ did not respond to EDTA treatment (**Figure 3**). These data suggest that the zinbo-5 cellular zinc pool is responsive to extracellular zinc levels. The regenerated zinc pool created by EDTA treatment remained responsive to a second zinc challenge (Supplementary Figure S1).

As shown in **Figure 3** cells pre-cultured in the presence of zinc store this cation internally, whilst cells pre-cultured in the presence of low zinc exhibit low initial zinbo-5 fluorescence following overnight culture. Therefore we next assessed zinc assimilation by *C. albicans*. **Table 1** shows that, following 18 h culture in the presence of 10 μM Zn^2+^, *C. albicans* take up half of the zinc from the culture medium. However, when these Zn^2+^-rich cells were subsequently pulsed with 5 μM Zn^2+^ for 20 min, they assimilated only 0.2 μM (±0.3 μM) Zn^2+^. In contrast, cells pre-cultured under lower Zn^2+^ conditions (SD + 1 μM Zn^2+^) assimilated more than half (2.7 μM) of exogenous Zn^2+^ during the 20 min pulse. These assimilation data indicate that cells from a zinc-rich preculture predominantly mobilize intracellular zinc reserves, as very little if any extracellular zinc is taken up during the treatment period. On the other hand, it would appear that cells from a zinc-poor preculture assimilate zinc during the 20 min treatment period, and that this freshly acquired zinc pool is subsequently mobilized to the ER following EDTA treatment.

**Table 1:** Zinc uptake during growth and zinc pulse.

	Zinc uptake from media during growth [μM]	Zinc uptake from 20 min 5 μM Zn^2+^ pulse [μM]
SD+1	–	2.7 ± 0.3
SD+10	5.0 ± 0.2	0.2 ± 0.3


*Uptake was measured as the decrease in zinc concentration in the media or buffer. Cells were cultured in minimal media containing 1 μM Zn^*2*+^ (SD+1) or 10 μM Zn^*2*+^ (SD+10). Standard deviation indicated from n = 3. “–” = not determined.*

In order to test the specificity of this response, cells from an SD + 1 μM Zn^2+^ culture were pre-treated with Zn^2+^, Cu^2+^, Ni^2+^, Co^2+^, Fe^2+^, Cd^2+^, Mg^2+^, or Ca^2+^, and then subsequently challenged with EDTA. Supplementary Figure S2 shows that only Zn^2+^ pretreatment resulted in a subsequent EDTA-induced increase in zinbo-5 signal. Therefore, of the divalent cations tested, our observed intracellular zinc fluxes are specific to Zn^2+^.

**FIGURE 4:**
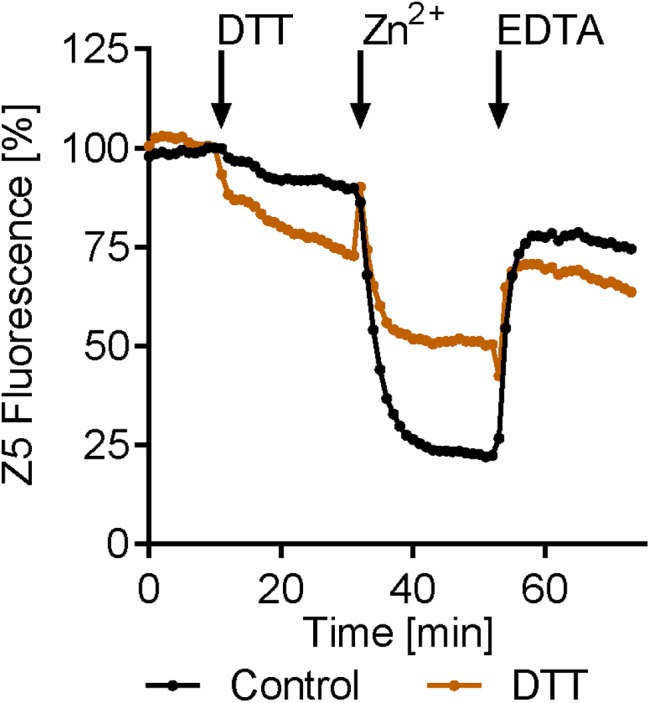
Endoplasmic reticulum (ER) stress modifies intracellular zinc release. Zinbo-5 (Z5) fluorescence was measured in cells pre-cultured in SD media containing 10 μM Zn^2+^. DTT was added for 20 min (stimuli) before 20 μM zinc was added for 20 min (Zn^2+^). Finally EDTA (100 μM) was added for 20 min *n* = 3.

The above data show that exogenous Zn^2+^ and EDTA can, respectively, stimulate the release and reentry of Zn^2+^ from and to the zinbo-5 compartment. It is possible that these events may be analogous to the stimulated release of ER zinc observed in mammalian cells (Taylor et al., 2012). Therefore the effect of ER stress on zinc release was tested. Cells were treated with the ER stress inducer DTT for 20 min. This treatment itself decreased zinbo-5 fluorescence by 27%, compared to 10% for the control (**Figure 4**). Subsequent addition of Zn^2+^ to the DTT-treated cells only decreased the zinbo-5 fluorescence to 50% of the initial signal, while control cells decreased by 78%. These data show that ER perturbation impacts the intracellular Zn^2+^ pool and supports our microscopy observations that zinbo-5 reports on ER-associated zinc (**Figures 1**, **2**).

**FIGURE 5:**
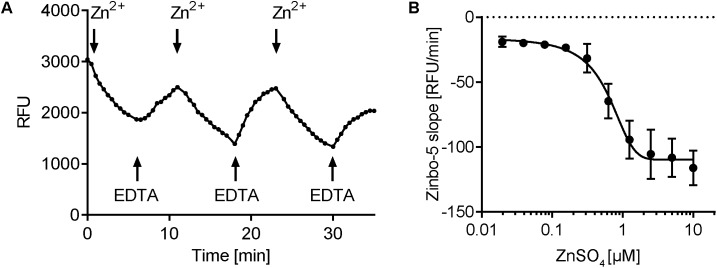
Zinbo-5 fluorescence changes upon extracellular zinc addition and chelation. **(A)** zinbo-5 fluorescence within cells in response to addition of Zn^2+^ and EDTA. Addition at the indicated arrows in the following order: 5 μM Zn^2+^, 10 μM EDTA, 20 μM Zn^2+^, 40 μM EDTA, 80 μM Zn^2+^, and 160 μM EDTA. **(B)** Titration of extracellular addition of ZnSO_4_. The slope was calculated in the first 10 min after zinc addition. Error bars indicate standard deviation. The EC_50_ value was calculated to 0.53 ± 0.19 μM ZnSO_4_ (*n* = 3).

As ER-released zinc serves as an intracellular second messenger in mammalian cells (Yamasaki et al., 2007), we next questioned the robustness of the response in fungal cells. Cells were repeatedly challenged with increasing concentrations of Zn^2+^ and EDTA. **Figure 5A** shows that the zinbo-5 zinc pool remained responsive to three sequential treatments. As *C. albicans* is both a human commensal and pathogen, we next sought to determine whether or not the observed intracellular zinc fluxes were physiologically relevant. To determine how much extracellular zinc is required to initiate intracellular zinc release, a dilution series of Zn^2+^ was applied to cells pre-grown in SD + 100 μM Zn^2+^ media. The rate of zinbo-5 decline within the first 10 min was then calculated and the EC_50_ value was found to be 0.53 μM (±0.19 μM) (**Figure 5B**). Therefore, *C. albicans* intracellular zinc release is responsive to physiologically relevant concentrations of this cation, which have been reported to be 12–18 μM in plasma (Strachan, 2010).

### Cyclic AMP Pathway Activation Induces Intracellular Zinc Mobilization

**FIGURE 6:**
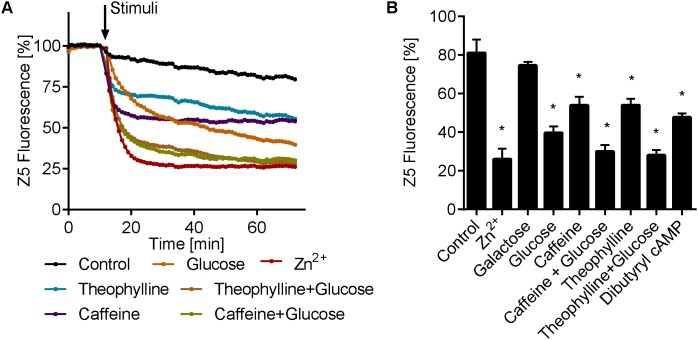
cAMP activation induce intracellular zinc release. **(A)** Time course of zinbo-5 (Z5) fluorescence following addition of cAMP pathway activators to cells grown in SD+10 media. **(B)** Same data as **A** showing the final zinbo-5 fluorescence after 1 h incubation with stimuli. Error bars represent standard deviation for *n* = 3–5. “^∗^” indicate *p* < 0.01 in one-way ANOVA compared to control. The following concentrations were used: 25 μM Zn^2+^, 0.25% glucose and galactose, 10 mM caffeine, 9.5 mM theophylline and 10 mM dibuturyl cAMP.

Our data suggest that, analogous to mammalian cells, the fungus *C. albicans* also triggers rapid intracellular zinc flux in response to extracellular zinc. We next questioned whether other environmental signals could also trigger intracellular zinc release. The cyclic AMP/protein kinase A (cAMP/PKA) pathway integrates multiple nutrient signals; moreover, we have previously shown that *C. albicans* rapidly activates that cAMP pathway in response to glucose, and that this cAMP spike is negatively regulated by the phosphodiesterase Pde1 (Wilson et al., 2010). We therefore pre-cultured *C. albicans* in SD + 10 μM Zn^2+^ to generate the intracellular zinc pool and then stimulated the cells with glucose. **Figure 6** shows that glucose stimulation also resulted in a rapid decrease in zinbo-5 fluorescence. In contrast, galactose stimulation had no effect. To test whether this effect was cAMP/PKA dependent, we simultaneously treated the cells with the cAMP agonist glucose and the broad spectrum phosphodiesterase (PDEase) inhibitors theophylline or caffeine. These combinations of cAMP/PKA pathway activation resulted in a much stronger decrease in zinbo-5 fluorescence. Finally, the cell permeable cAMP analog dibutyryl cAMP also triggered zinc release at levels comparable to PDEase inhibition (**Figure 6B**). These data indicate that cAMP/PKA pathway activation triggers intracellular zinc release in *C. albicans*.

### Inhibition of the cAMP Pathway Inhibits Glucose-Induced Intracellular Zinc Release

**FIGURE 7:**
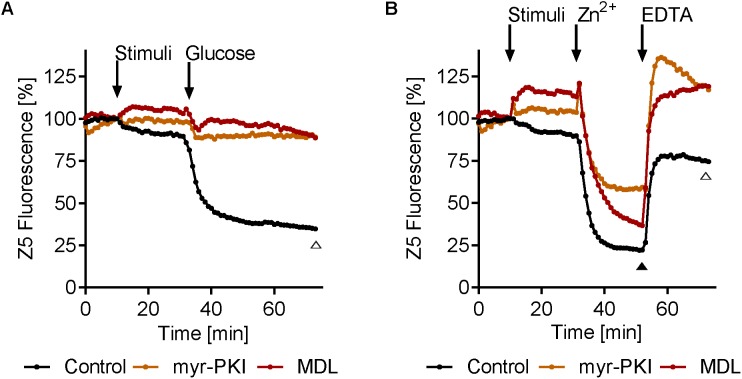
Inhibition of cAMP signaling inhibits glucose-induced zinc mobilization. **(A)** Adenylate cyclase (AC) inhibitor MDL12330 (MDL, 417 μM) or PKA inhibitor myr-PKI (25 μM) was added for 20 min (stimuli) before addition of 0.25% glucose. **(B)** MDL12330 (MDL, 417 μM) and myr-PKI (25 μM) was added for 20 min (stimuli) before 20 μM zinc was added for 20 min (Zn^2+^). Finally 100 μM EDTA was added for 20 min. Open triangle (Δ) indicate *p* < 0.01 in one-way ANOVA test for both myr-PKI and MDL compared to control at the specified time point, while closed triangle (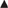) indicate *p* ≤ 0.01 in one-way ANOVA test for only myr-PKI *n* = 3–6.

Next we tested the effect of cAMP/PKA pathway inhibition on intracellular zinc release. MDL12330 and myr-PKI inhibit the adenylate cyclase (AC) and PKA, respectively (Castilla et al., 1998; Jain et al., 2003). Both compounds resulted in a modest increase in zinbo-5 signal, in line with PKA activity negatively regulating the intracellular zinc pool (**Figures 7A,B**). Interestingly, cAMP/PKA inhibition fully blocked glucose- (**Figure 7A**), but not Zn^2+^-induced (**Figure 7B**) zinc release. However, treatment with myr-PKI moderately inhibited Zn^2+^ release upon treatment with extracellular Zn^2+^ and both myr-PKI and MDL12330 enhanced subsequent EDTA-induced Zn^2+^ reentry to the zinbo-5 compartment (**Figure 7B**). These results indicate that glucose activates zinc release through cAMP signaling, while Zn^2+^ stimulation can at least partially bypass the cyclic AMP/PKA pathway.

## Discussion

In humans, zinc acts as both an intercellular (e.g., neurotransmission) (Sensi et al., 2009) and an intracellular signaling molecule, analogous to well-known second messengers such as Ca^2+^ and cyclic AMP (Fukada et al., 2011).

While its role as an essential micronutrient in fungi is relatively well established, a function for zinc in cell signaling has not, to the best of our knowledge, been described in the fungal kingdom. The only well-described zinc sensor in fungi is the transcription factor Zap1. In the model yeast, *S. cerevisiae*, Zap1 positively regulates the expression of zinc uptake genes as cellular zinc becomes limited (Wilson and Bird, 2016) and the *C. albicans* ortholog (Zap1/Csr1) appears to have a similar role (Ballou and Wilson, 2016).

Here we demonstrate rapid changes in fungal intracellular zinc pools in response to two, apparently distinct environmental signals. When cells were cultured overnight in the presence of (≥10 μM) Zn^2+^, they assimilated (**Table 1**) and stored the cation in the internal membrane system, including the ER – the zinbo-5 detectable zinc pool (**Figures 1**–**3**). When stimulated with external zinc, these cells immediately began to mobilize this intracellular pool (**Figure 3**), yet did not assimilate significant levels of zinc from the medium over the 20 min treatment (**Table 1**). It is possible that the rapid drop in zinbo-5 signal observed here represents direct release of zinc into the cytoplasm, which may act in itself as a second messenger, as has been observed in mammalian cells (Yamasaki et al., 2007).

This indicates that the observed intracellular response to extracellular zinc is likely to be Zap1-independent. Primarily, mobilization occurred immediately following zinc stimulation (**Figure 3**), which is too rapid to be the result of transcriptional changes; secondly, virtually none of the added zinc actually entered the fungal cell (**Table 1**). Finally, when these cells were treated with the cell impermeable zinc chelator EDTA, zinc was rapidly re-compartmentalized within the ER (**Figure 3**). These three lines of evidence suggest the existence of an extracellular zinc sensing system in *C. albicans*.

Animals encode a plasma membrane G-protein coupled plasma membrane receptor called Gpr39/ZnR, which in humans has been reported to sense extracellular zinc and trigger intracellular signaling events (Hershfinkel et al., 2001; Sunuwar et al., 2017). Although fungi do not share an ortholog, it is interesting to note that Gpr39-mediated signaling was also responsive to micromolar levels of extracellular zinc and did not involve zinc import (Hershfinkel et al., 2001).

Recently, the *S. cerevisiae* plasma membrane protein Zrt1 has been identified as a fungal transceptor, that in addition to its well-defined role in high affinity zinc assimilation, can also sense extracellular zinc. Intriguingly, addition of zinc to zinc starved cells resulted in rapid (1–2 min) Zrt1-dependent trehalase activation – a classical target of the PKA pathway in yeast (Schothorst et al., 2017).

**FIGURE 8:**
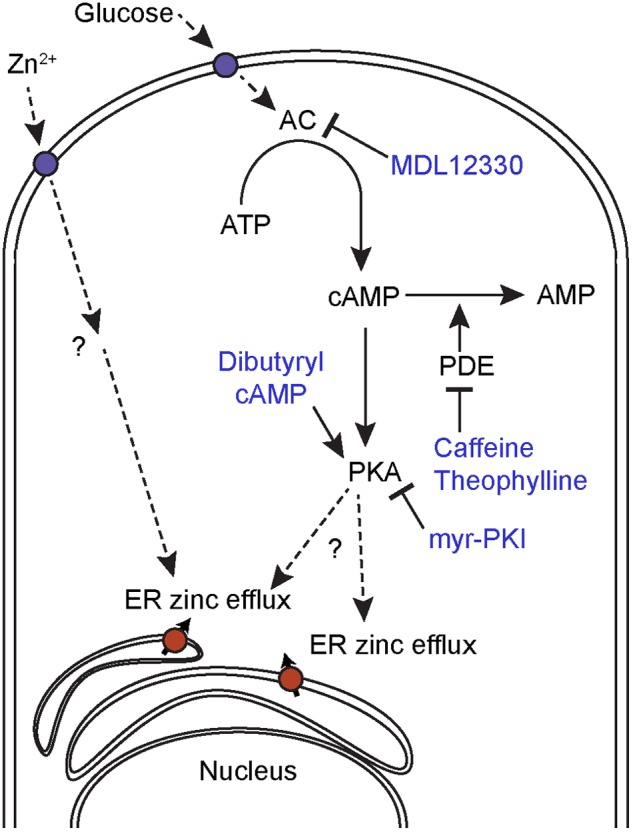
Proposed mechanism for intracellular zinc mobilization. Glucose induces cAMP-PKA-dependent intracellular zinc release and can be inhibited by the AC inhibitor MDL12330 or the PKA inhibitor myr-PKI. The cAMP analog, dibutyryl cAMP and PDE inhibitors, also induces intracellular zinc release. Extracellular Zn^2+^ induces ER zinc efflux through a cAMP-PKA independent mechanism.

At this stage it is unclear whether *C. albicans* responds to extracellular zinc via a GPCR like in animals, or via a transceptor like *S. cerevisiae* or an as yet unknown mechanism (**Figure 8**).

Release of intracellular pools in response to stimulation with extracellular zinc has been observed previously. Taylor et al. (2012) reported that treatment of mammalian cells with extracellular zinc or with epidermal growth factor resulted in ER zinc efflux. However, subsequent studies suggest that the zinc flux observed may have been due to ionophore-mediated uptake of zinc from the culture medium (Hessels and Merkx, 2015). We did not use an ionophore in the current study.

In addition to extracellular zinc, we also observed cAMP/PKA-dependent changes in the intracellular zinc pool. Stimulation of cells with the cAMP/PKA agonist glucose resulted in rapid mobilization, and this effect was amplified by the addition of PDEase inhibitors. PDEase inhibitors stimulate the PKA pathway by preventing the hydrolysis of cAMP. Likewise, treatment of cells with the cAMP analog, dibutyryl cAMP had a similar effect. In contrast, inhibition of AC (MDL12330) or PKA activity (myr-PKI) prevented glucose-induced zinc flux. These four lines of evidence suggest that cAMP/PKA activation generates rapid changes in intracellular zinc localization. At this stage it is not clear whether the released zinc acts as second messenger in fungi, as has been shown for mammalian cells.

Interestingly, it would appear that extracellular zinc and cAMP/PKA pathway activation represent distinct mediators of intracellular zinc flux as the zinc-induced flux was not blocked by AC or PKA inhibitors. We therefore propose a model whereby glucose induce ER zinc efflux to the cytoplasm mediated by cAMP/PKA signaling, whereas exogenous zinc induce ER zinc efflux in a cAMP/PKA independent manner (**Figure 8**).

In summary, this study is the first report of agonist-induced changes in intracellular zinc distribution in a fungal species. Intriguingly, this intracellular zinc flux is triggered by changes in either environmental zinc or glucose and we define a novel role for cyclic AMP/PKA signaling in controlling the intracellular zinc pool.

## Author Contributions

LK, A-MW, DW, and AF conceived the study, acquired (LK), analyzed and interpreted the data, drafted the manuscript and critically revised the manuscript. All authors approved the final submitted version and agreed to accountability for all aspects of the work.

## Conflict of Interest Statement

LK and A-MW are or were employees of Pcovery ApS. The other authors declare that the research was conducted in the absence of any commercial or financial relationships that could be construed as a potential conflict of interest.
