# Supplementary material for: Cyclic AMP Pathway Activation and Extracellular Zinc Induce Rapid Intracellular Zinc Mobilization in Candida albicans
Source: Front Microbiol. 2018 Mar 21;9:502. doi: 10.3389/fmicb.2018.00502 (PMC5871664; doi:10.3389/fmicb.2018.00502)
Supplement: Supplementary file 1 [file Data_Sheet_1.docx]

Supplementary Material

Cyclic AMP pathway activation and extracellular zinc induce rapid intracellular zinc mobilization in *Candida albicans*

Lasse Kjellerup^1,2^, Anne-Marie Lund Winther^2^, Duncan Wilson^3#^, Anja Thoe Fuglsang^1#*^

^1^Department of Plant and Environmental Sciences, University of Copenhagen, Frederiksberg, Denmark

^2^Pcovery ApS, Copenhagen, Denmark

^3^Aberdeen Fungal Group, MRC Centre for Medical Mycology, University of Aberdeen, Institute of Medical Sciences, Aberdeen, United Kingdom

**#** Co-senior author.

*** Correspondence:** Anja Thoe Fuglsang: atf@plen.ku.dk

## Supplementary Figures





**Supplementary Figure S1 – Environmental Zn^2+^ induces dynamic changes in the cellular zinc pool.** Cells were grown in SD media containing 1 µM Zn^2+^ (SD+1), 10 µM Zn^2+^ (SD+10) or 100 µM Zn^2+^ (SD+100). Zinbo-5 was added and the zinbo-5 fluorescence determined following 10 min of incubation with probe. Subsequently 5.7 µM zinc (Zn^2+^) was added for 30 min, 50 µM EDTA was then added for 20 min and, finally, 111 µM zinc (Zn^2+^) was added for 20 min. Error bars indicate standard deviation for *n* = 3.


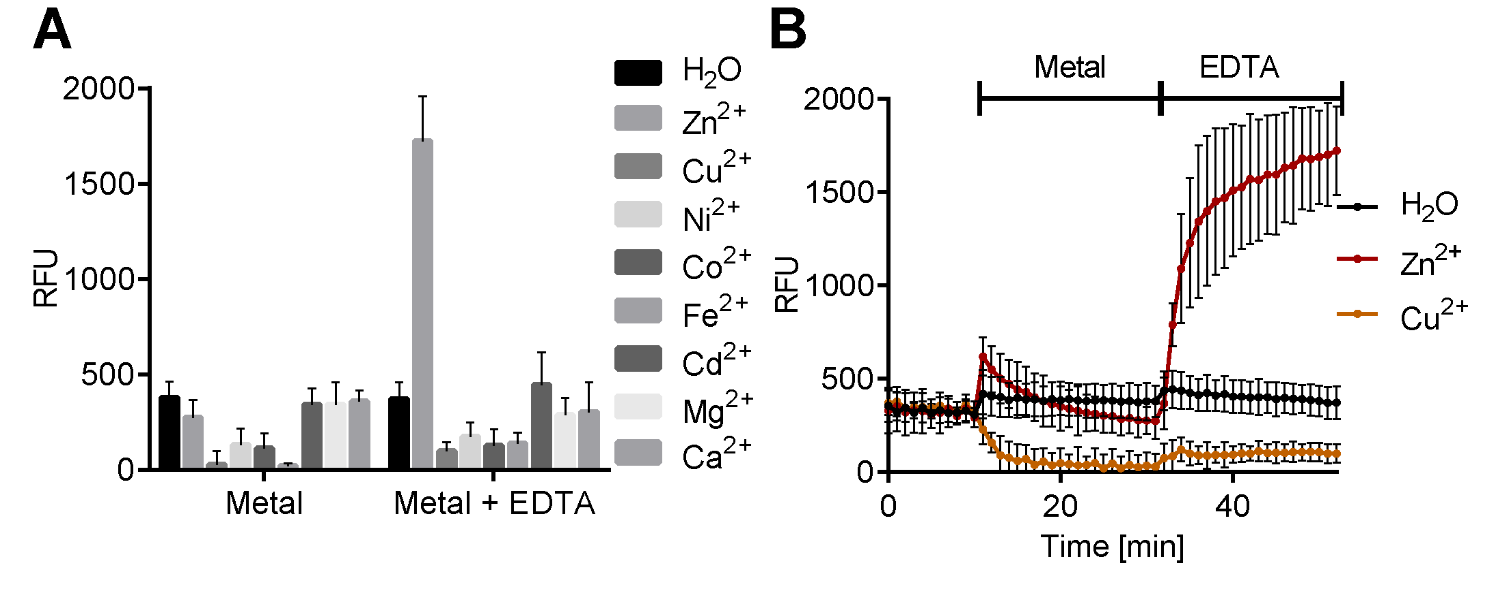


**Supplementary Figure S2 – Zinbo-5 only reports on zinc in metal uptake assay**. **A**) The indicated metal at 23 µM was added to zinbo-5 stained cells from a SD+1 pre-culture for 20 min. followed by 250 µM EDTA for 20 min. **B**) Time curves for selected data from **A**. Error bars indicate standard deviation for *n* = 3.
